# Supplementary material for: Causal Relationship between Aging and Anorexia Nervosa: A White-Matter-Microstructure-Mediated Mendelian Randomization Analysis
Source: Biomedicines. 2024 Aug 16;12(8):1874. doi: 10.3390/biomedicines12081874 (PMC11351342; doi:10.3390/biomedicines12081874)
Supplement: Supplementary file 1 [file biomedicines-12-01874-s001.zip › Supplementary Figure/Figure S4/Singe SNP plot of MR analysis for TL to ubm-b-1821.pdf]

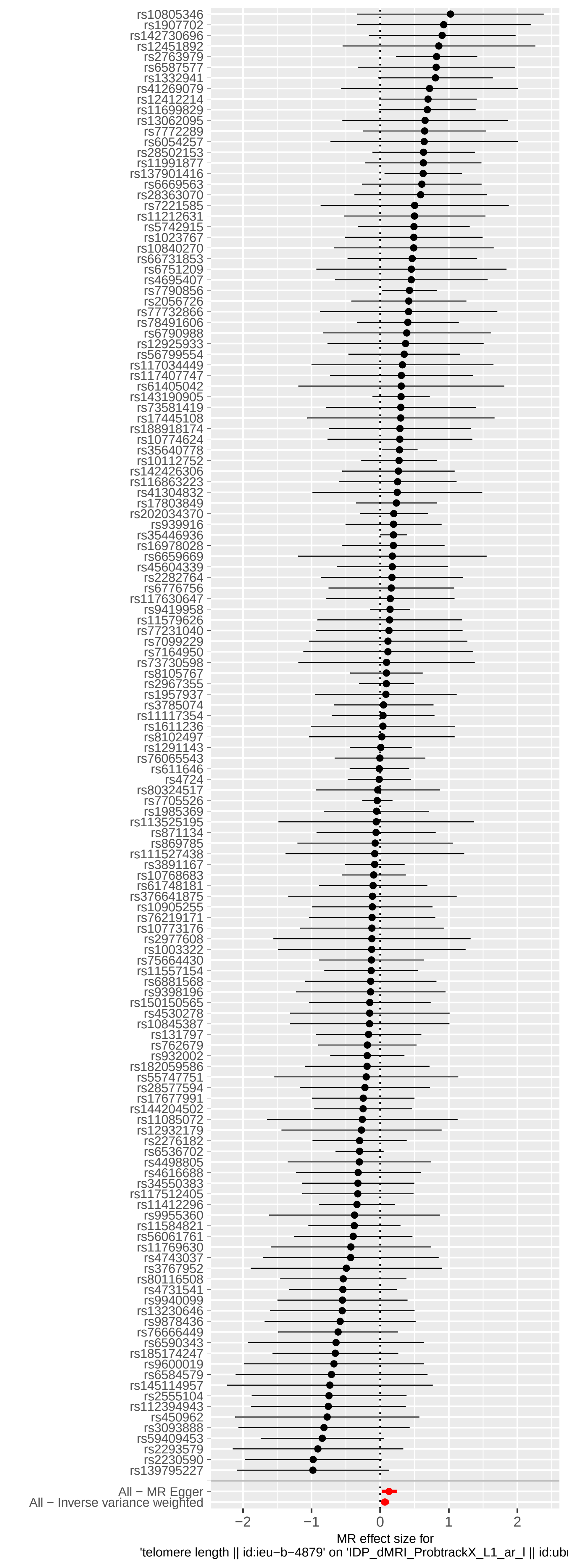

rs10805346
rs1907702
rs142730696
rs12451892
rs2763979
rs6587577
rs1332941
rs41269079
rs12412214
rs11699829
rs13062095
rs7772289
rs6054257
rs28502153
rs11991877
rs137901416
rs6669563
rs28363070
rs7221585
rs11212631
rs5742915
rs1023767
rs10840270
rs66731853
rs6751209
rs4695407
rs7790856
rs2056726
rs77732866
rs78491606
rs6790988
rs12925933
rs56799554
rs117034449
rs117407747
rs61405042
rs143190905
rs73581419
rs17445108
rs188918174
rs10774624
rs35640778
rs10112752
rs142426306
rs116863223
rs41304832
rs17803849
rs202034370
rs939916
rs35446936
rs16978028
rs6659669
rs45604339
rs2282764
rs6776756
rs117630647
rs9419958
rs11579626
rs77231040
rs7099229
rs7164950
rs73730598
rs8105767
rs2967355
rs1957937
rs3785074
rs11117354
rs1611236
rs8102497
rs1291143
rs76065543
rs611646
rs4724
rs80324517
rs7705526
rs1985369
rs113525195
rs871134
rs869785
rs111527438
rs3891167
rs10768683
rs61748181
rs376641875
rs10905255
rs76219171
rs10773176
rs2977608
rs1003322
rs75664430
rs11557154
rs6881568
rs9398196
rs150150565
rs4530278
rs10845387
rs131797
rs762679
rs932002
rs182059586
rs55747751
rs28577594
rs17677991
rs144204502
rs11085072
rs12932179
rs2276182
rs6536702
rs4498805
rs4616688
rs34550383
rs117512405
rs11412296
rs9955360
rs11584821
rs56061711
rs11769630
rs4743037
rs3767952
rs80116508
rs4731541
rs9940099
rs13230646
rs9878436
rs76666449
rs6590343
rs185174247
rs9600019
rs6584579
rs145114957
rs2555104
rs112394943
rs450962
rs3093888
rs59409453
rs2293579
rs2230590
rs139795227
All − MR Egger
All − Inverse variance weighted
−2
−1
0
1
2
MR effect size for
'telomere length || id:ieu−b−4879' on 'IDP_dMRI_ProbtrackX_L1_ar_l || id:ub
